# *BRCA1* promoter hypermethylation on circulating tumor DNA correlates with improved survival of patients with ovarian cancer

**DOI:** 10.1002/1878-0261.13108

**Published:** 2021-10-12

**Authors:** Maha Elazezy, Katharina Prieske, Lan Kluwe, Leticia Oliveira‐Ferrer, Sven Peine, Volkmar Müller, Linn Woelber, Barbara Schmalfeldt, Klaus Pantel, Simon A. Joosse

**Affiliations:** ^1^ Department of Tumor Biology University Medical Center Hamburg‐Eppendorf Hamburg Germany; ^2^ Department of Gynecology and Gynecologic Oncology University Medical Center Hamburg‐Eppendorf Hamburg Germany; ^3^ Mildred Scheel Cancer Career Center HaTriCS4 University Medical Center Hamburg‐Eppendorf Hamburg Germany; ^4^ Department of Oral and Maxillofacial Surgery University Medical Center Hamburg‐Eppendorf Hamburg Germany; ^5^ Department of Transfusion Medicine University Medical Center Hamburg‐Eppendorf Hamburg Germany

**Keywords:** *BRCA1*, ctDNA, liquid biopsy, MS‐qPCR

## Abstract

Methylation of the *BRCA1* promoter is an epigenetic gene expression regulator and is frequently observed in ovarian cancer; however, conversion of methylation status is thought to drive disease recurrence. Therefore, longitudinal monitoring of methylation status by liquid biopsy in cell‐free DNA may be a predictive marker. In total, 135 plasma samples were collected from 69 ovarian cancer patients before and during systemic treatment. Our liquid biopsy assay could detect down to a single molecule of methylated DNA in a high background of normal DNA (0.03%) with perfect specificity in control samples. We found that 60% of the cancer patients exhibited *BRCA1* promoter hypermethylation at one point, although 24% lost hypermethylation during treatment. Multivariate survival analyses indicate that relapses are independent events and that hypermethylation and methylation conversion are independently correlated to longer relapse‐free survival. We present a highly sensitive and specific methylation‐specific quantitative PCR‐based liquid biopsy assay. *BRCA1* promoter hypermethylation is frequently found in ovarian cancer and is often reversed upon recurrence, indicating the selection of therapy‐resistant clones and unfavorable clinical outcome.

## Graphical abstract

In patients with ovarian cancer (OC) undergoing platinum‐based chemotherapy and PARP‐inhibitor treatment, *BRCA1* deficiency is used as major predictive marker for estimating response to therapy. Here, we present a highly sensitive and specific liquid biopsy assay that assesses *BRCA1* gene promoter hypermethylation on circulating tumor DNA (ctDNA) isolated from blood plasma. Our results suggest that hypermethylation and methylation conversion of the *BRCA1* promoter in ctDNA are independently correlated to longer progression‐free survival of patients with OC.

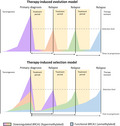

**Abbreviations**
*BRCA1*
: breast cancer 1
cfDNA: cell‐free DNA
CI: confidence interval
ctDNA: circulating tumor DNA
FIGO: International Federation of Gynecology and Obstetrics
HR: hazard ratio
MS‐qPCR: methylation‐specific quantitative polymerase chain reaction

## 1 Introduction

Germline and somatic mutations in *BRCA1* have been identified in approximately one‐third of ovarian carcinomas, and their presence is highly predictive of primary platinum and PARP (Poly‐ADP‐Ribose‐Polymerase) inhibitors sensitivity and favorable progression‐free and overall survival [1, 2, 3, 4]. Hypermethylation of the *BRCA1* promoter leads to downregulation of *BRCA1* mRNA expression [5, 6], resulting in defective homologous recombination characterized by typical chromosomal aberrations seen in *BRCA1* mutation carriers [6, 7]. Although hypermethylation of the *BRCA1* promoter has been shown in xenografts to predict response to PARP inhibitors as well [8], data on patient survival are still unclear [9]. Recently, we showed that the *BRCA1* promoter is frequently hypermethylated in ovarian cancer tissue, but the methylation status is often lost in recurrent disease, suggesting a potential resistance mechanism either through therapy‐induced cancer evolution or by clonal selection [10, 11, 12]. Thereby, the detection and monitoring of *BRCA1* promoter hypermethylation may have an important impact on the clinical management of ovarian cancer patients without *BRCA1* mutation [8]. Precision medicine in oncology may be achieved through the diagnostic method ‘liquid biopsy’ as has been shown in several cancer entities [13, 14, 15, 16]. This method utilizes the detection of biomarkers in blood or other body liquids for prognostic and predictive purposes and has several advances over using tissue alone [15]. Circulating tumor DNA (ctDNA) are cell‐free DNA (cfDNA) fragments released into the circulation by tumor cells and can provide direct information about the methylomic make‐up of the tumor currently present in the patient [17, 18]. The purpose of this study was to develop a liquid biopsy assay that could determine the methylation status of the *BRCA1* promoter to be able to monitor hypermethylation of the *BRCA1* promoter and investigate its clinical significance as a predictive biomarker in ovarian cancer patients. We considered two models of cancer progression leading to therapy resistance: cancer evolution driven by therapy pressure and, secondly, selection and survival of tumor clones originating from the primary tumor (illustrated in Fig. S1).

## 2 Materials and methods

### 2.1 Sample collection

In this prospective study, 69 ovarian cancer patients treated during 2015–2020 were included. Selection criteria were primary or recurrent, high‐grade serous ovarian cancer with platinum‐based first‐line therapy (carboplatin, *n* = 68; cisplatin, *n* = 1) (Table S1). Blood sampling was performed before treatment, at relapse before therapy change, and/or during the course of therapy. Additionally, 69 healthy, age‐matched women were included as controls. This study was approved by the local ethical board (ethical approval number: PV5392) in accordance with the declaration of Helsinki, all participants enrolled into this study gave written informed consent. The patients' demographic statistics are described in Table 1.

**Table 1:** Demographic statistics. *P*‐values were calculated using the *G*‐test with Williams' correction for count data and ANOVA for continuous data. The study cohort was divided by the methylation status of the *BRCA1* promoter of the patients: hypermethylated detected in all blood samples (ME1), no methylated detected in any of the blood samples (M0), and methylation status changed during the course of treatment from positive to negative (MEc).

	ME1	ME0	MEc	Total	*P*‐value
Mean age (years)	56.7	60.8	58.0	58.3	0.60
FIGO stage					
I–IIIB	2	2	3	7	0.39
IIIC	19	16	4	37	
IV	9	5	3	17	
Grade					
G2	4	2	1	7	0.92
G3	30	22	9	32	
T‐stage					
T1	1	2	1	4	0.41
T2	1	3	0	4	
T3	28	15	7	50	
N‐stage					
N0	6	4	1	11	0.79
N1	15	10	4	29	
Nx	4	3	0	7	
Residual tumor					
No (macroscopic complete resection)	18	12	4	34	0.93
Yes	15	8	3	26	
Lymphatic invasion					
L0	6	7	1	14	0.29
L1	22	11	7	40	
Venous invasion					
V0	24	15	7	37	0.99
V1	3	2	1	6	
PARP inhibitor treatment					
Yes	6	5	4	15	0.40
No	25	22	6	53	
Germline *BRCA1*					
Mutated	5	6	2	13	0.64
Wild‐type/Unknown	29	18	8	55	

### 2.2 Cell‐free DNA isolation and bisulfite conversion

Peripheral blood of all women was collected in EDTA containing tubes and processed within 1 h. Blood was centrifuged at 360 ***g*** for 20 min, and plasma was centrifuged again at 5087 ***g*** for 10 min. cfDNA was isolated and treated with bisulfite by the full automated InviGenius^®^ Plus instrument with the InviMag^®^ Free Circulating DNA Kit/IG (cat.no. 2439320400; Invitek Molecular, Berlin, Germany) and InviMag^®^ Bisulfite Conversion Kit/IG (cat.no. 3030200100; Invitek Molecular). cfDNA quantification and fragment size distribution were assessed using the Agilent 2200 TapeStation High Sensitivity D5000 and Qubit^®^ 2.0 Fluorometer dsDNA HS Assay Kits (Thermo Fisher Scientific, Eugene, OR, USA) according to the manufacturers' instructions.

### 2.3 Methylation‐specific, quantitative real‐time PCR

BRCA1 promoter methylation status in cfDNA was assessed after bisulfite conversion using methylation‐specific primers as before [10] with slight adjustments to the PCR protocol. The sequences of the primers for amplifying the 1543‐ to 1617‐bp region (fragment length, 75 bp) of the *BRCA1* (GenBank U37574.1) promoter in case of methylation were 5′‐TCGTGGTAACGGAAAAGCGC‐3′ (sense) and 5′‐AAATCTCAACGAACTCACGCCG‐3′ (antisense). The primers for amplifying the 1536–1621 bp region (fragment size), 86 bp of the wild‐type *BRCA1* promoter were 5′‐TTGGTTTTTGTGGTAATGGAAAAGTGT‐3′ (sense) and 5′‐CAAAAAATCTCAACAAACTCACACCA‐3′ (antisense). Methylation‐specific, quantitative real‐time PCR (MS‐qPCR) was performed in 15 μL reaction volume containing 1× PCR buffer (1.5 mm MgCl_2_, 10 mm Tris/HCl of pH 8.3, 50 mm KCl), 100 µm of each dNTP, 0.2 µm of each primer, 0.1× SYBR green, 0.25 µg BSA, and 0.5 U JumpStart™ Taq DNA Polymerase (cat no. D9307; Merck, Darmstadt, Germany). The MS‐qPCR reaction was applied in a CFX96 Touch™ Real‐Time PCR Detection System (Bio‐Rad Laboratories GmbH, Feldkirchen, Germany). The PCR conditions were as follow: initial denaturation at 94 °C for 1 min, followed by 40 amplification cycles of 94 °C for 30 s, 63 °C for 30 s, and 72 °C for 30 s, a final extension at 72 °C for 1 min, and a melting curve of 65.0–95.0 °C with increments of 0.5 °C every 5 s.

### 2.4 Liquid biopsy assay establishment

The sensitivity and specificity of our MS‐qPCR‐based liquid biopsy assay were assessed by testing serial dilutions of methylated reference DNA ‘Human HCT116 DKO Methylated DNA’ from Zymo Research (Freiburg, Germany) and unmethylated reference DNA isolated from a pool of healthy donors (cat. No. G1521; Promega, Walldorf, Germany). The DNA dilution mix ranged from 100 to 1 genome copies in a background of wild‐type unmethylated DNA equivalent to 3000 genome copies. Methylated and unmethylated reference DNA was fragmented using Bioruptor^®^ Plus sonication system for 20 cycles (20 s on, 30 s off) to a length similar to that of patients' cfDNA, followed by bisulfite treatment. Quantitative and qualitative detection of the methylation status was determined via melting curve analysis of the MS‐qPCR amplified products, as well as from agarose gel electrophorese for confirmation.

### 2.5 cfDNA sequencing

The MS‐qPCR products were separated by electrophoresis on a 3% agarose gel stained with ethidium bromide. The corresponding bands were cut out of the gel and purified using NucleoSpin Gel and PCR Clean‑up for QIAcube (cat. No. 15116456; Macherey‐Nagel, Dueren, Germany). Next, Sanger sequencing was performed to confirm the methylation status of the *BRCA1* promoter as before [10]. Ideally, 15 ng of the purified PCR product was used for sequencing with the same reverse primer used for the MS‐qPCRs. PCR sequencing was performed using Big Dye™ Terminator v1.1 Cycle Sequencing Kit (cat. No. 4337451; Applied Biosystems, Foster City, CA, USA) and analyzed by 3130 Genetic Analyzer (Thermo Fisher). CpG islands of bisulfite sequences were aligned and analyzed using quma [19].

### 2.6 Statistical analysis

Statistical analyses were performed using r (R Foundation for Statistical Computing, version 3.6.3, Vienna, Austria) and In‐Silico Online, version 2.1.2 [20]. Because ovarian cancer patients can relapse multiple times during the course of their disease, analysis based only on the first event time cannot be used to examine the effect of the risk factors on the number of recurrences over time [21]. Therefore, progression‐free survival analyses were performed using Kaplan–Meier curves for recurrent events using two multivariate models. The first model tests the correlation between methylation status and the gap times between successive progression events. This model assumes that the occurrence of an event is related to the previous event, however, this assumption only holds true if a relapse originates from the preceding tumor (illustrated in Fig. S1). The second model tests the correlation between methylation status and the time from initial diagnosis to each event of progression of disease independently for each patient. This model assumes that the occurrence of subsequent events are not correlated, and this assumption only holds true if all relapses originate from the primary tumor (illustrated in Fig. S1). The endpoints were progression and cancer‐related death according to remark [22]. The clinical variables (residual tumor, FIGO stage, grade, T‐stage, N‐stage, or treatment with PARP inhibitors) were compared to the methylation status of *BRCA1* promoter of ovarian cancer patients using ANOVA for continuous data and *P*‐values were calculated using the *G*‐test with Williams' correction for count data. The nonsignificant clinical variables (FIGO stage, T‐stage, N‐stage, and grade) were excluded from the multivariate analysis. The power of sample size was analyzed by pass, version 20.0.2 (NCSS LLC, East Kaysville, UT, USA).

## 3 Results

### 3.1 Liquid biopsy assay establishment

**Fig. 1:**
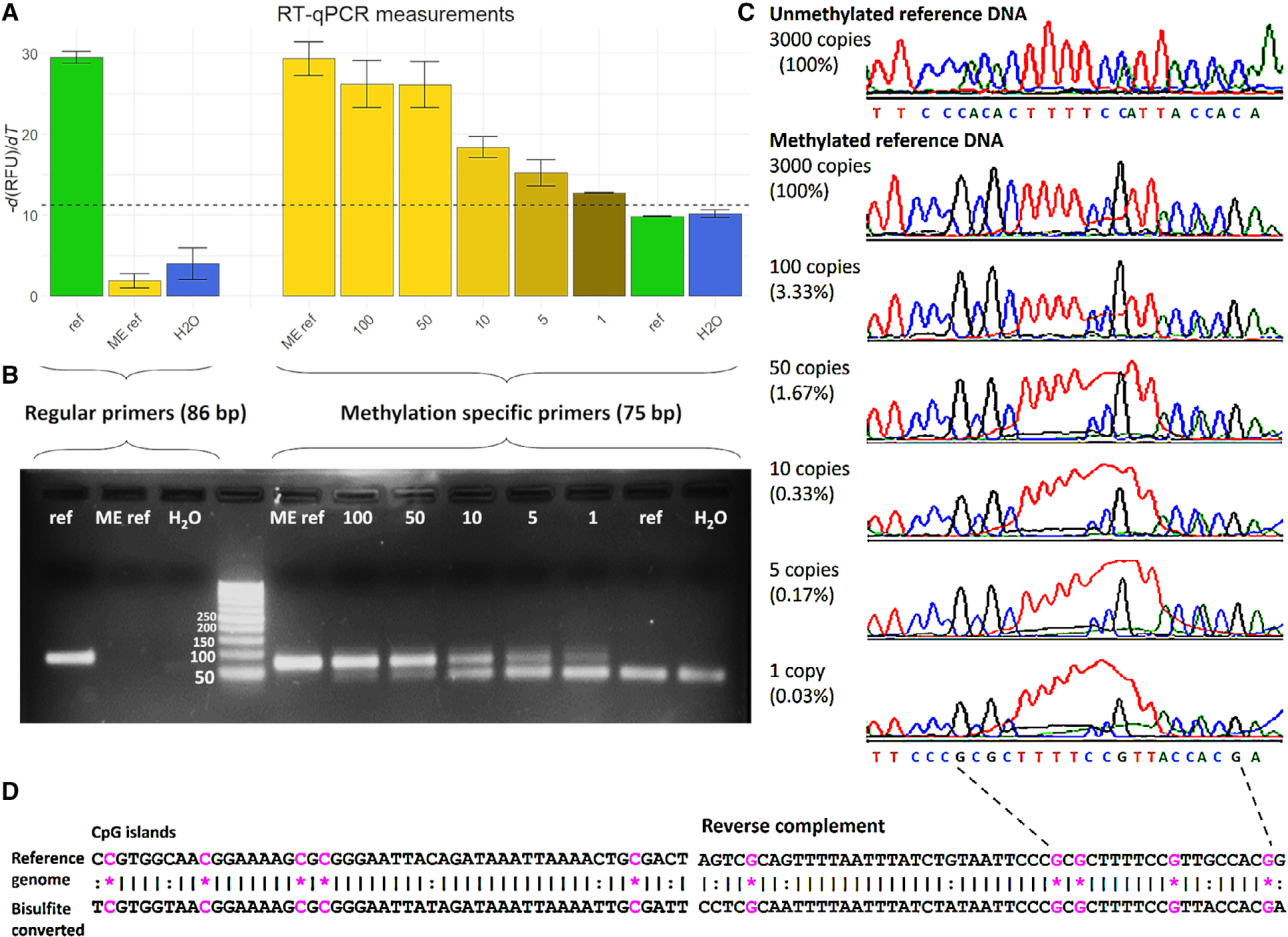
Measurement of methylated *BRCA1* promoter by MS‐qPCR. Two sets of primers were used to detect unmethylated *BRCA1* promoter DNA (regular primers, fragment length 86 bp) and methylated *BRCA1* promoter DNA (methylation‐specific primers, fragment length 75 bp). Sensitivity and specificity of both primer sets and PCR protocol were assessed using unmethylated human reference DNA (ref), methylated human reference DNA (ME ref), water (H_2_O), and a dilution series containing 100, 50, 10, 5, and 1 copy of methylated human reference DNA in a background of unmethylated human reference DNA with a total amount of 3000 copies of gDNA. (A) Average −d(RFU)/d*T* values measured in RT‐qPCR, error bars represent standard deviations (*n* = 3). (B) Gel electrophoreses photo of PCR amplified products. (C) Sanger sequencing results of the PCR amplified products. (D) Genome sequence of investigated CpG islands and sequence after bisulfite conversion and PCR amplification in case of methylation. Sequences outputted by Sanger sequencing are reversed complemented. *, methylated CpG site (pink); :, non‐CpG converted cytosine to thymine; |, matching base.

Sensitivity of detection of *BRCA1* gene promoter hypermethylation was tested in a dilution series of hypermethylated DNA mixed with wild‐type, nonmethylated DNA with a total amount of DNA equivalent to 3000 copies of gDNA (the average amount of DNA per mL plasma found in healthy individuals [23]). The dilution series of hypermethylated DNA consisted of 100% (3000 gDNA copies), 3.33% (100 gDNA copies), 1.67% (50 gDNA copies), 0.33% (10 gDNA copies), 0.17% (5 gDNA copies), and 0.03% (1 gDNA copy), and was measured in triplicate twice. Melting curve and gel electrophoresis analyses of the amplified products showed highly sensitive detection of all diluted hypermethylated DNA samples (Fig. 1A,B). The methylation status of MS‐qPCR products was confirmed using Sanger sequencing (Fig. 1C,D). These results demonstrated the ability to detect hypermethylated *BRCA1* gene promoter, down to a single molecule, in a background of 99.97% normal DNA.

### 3.2 Study cohort and sample material

**Fig. 2:**
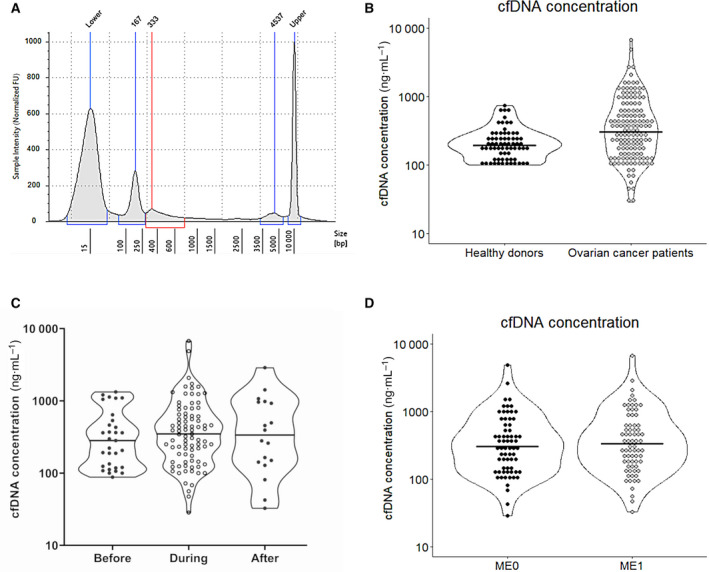
Isolated cfDNA. (A) Fragment size distribution of cfDNA obtained from a single ovarian cancer case. Peaks marked *Lower* and *Upper* signify the size standards used by the TapeStation to estimate the other three peaks. The two peaks labeled 167 and 333 bp represent DNA originating from apoptotic and necrotic cells [16], whereas the 4537 bp peak represents the gDNA from lysed leukocytes after blood sampling. (B) Violin plot showing the distribution of the cfDNA concentrations in ng·mL^−1^ plasma from blood samples obtained from healthy donors (*n* = 69) and ovarian cancer patients (*n* = 135). (C) Violin plot showing the cfDNA concentrations distribution in blood taken before (*n* = 31), during (*n* = 85), and after (*n* = 18) the systemic therapy. (D) Violin plot depicting the distribution of the cfDNA concentrations of ovarian cancer patients with (ME1; *n* = 61) or without (ME0 *n* = 71) hypermethylated BRCA1 promoter.

In total, 135 plasma samples from 69 advanced‐stage ovarian cancer patients were obtained; 111 multiple longitudinal blood samples were collected from 41/69 patients during the course of disease. The patients' mean age was 58.3 years (range: 31–89); the healthy donors' mean age was 56.2 years (range: 30–73). The median follow‐up was 39.3 months [95% confidence interval (CI): 25.0–49.6 months], starting from the time point of first diagnosis. The cfDNA fragment size in ovarian cancer patients was on average 166 bp (*s* = 17.2) and 338 bp (*s* = 48.3; Fig. 2A). The median concentration of total cfDNA obtained from all patients' blood samples at all time points was 306 ng·mL^−1^ plasma (range: 28.4–6750), whereas the median of cfDNA from a healthy donor was 192 ng·mL^−1^ plasma (range: 100–742; Fig. 2B). cfDNA concentration of ovarian cancer patients was significantly higher as compared to healthy controls (*P* = 0.0002, Wilcoxon rank sum test with continuity correction). No significant differences were observed between the median cfDNA concentrations of 282, 348, and 337 ng·mL^−1^ plasma of before, during, and after systemic therapy, respectively (*P* = 0.535, ANOVA; Fig. 2C). Out of 33 patients who were tested for germline mutations in the *BRCA1* gene, 12 tested positive and were therefore also analyzed separately.

### 3.3 *BRCA1* promoter hypermethylation in cfDNA

*BRCA1* promoter hypermethylation was detected during the course of the disease until the end of follow‐up in 46% (31/68) of patients, no methylation could be detected in 40% (27/68), and 15% (10/68) of patients converted from having hypermethylation to no methylation until the end of follow‐up; data of one patient could not be obtained (1/69). Two patients started with a negative methylation status, which was positive in subsequent cfDNA samples, and these patients were included into the methylation positive group (2/31). The methylation status of patients of whom only one plasma sample was obtained was assumed to remain stable, and the patients were grouped into either the methylation positive or negative group. The median of cfDNA concentrations in the methylation positive and negative ovarian cancer patients was 316 and 344 ng·mL^−1^ plasma (Fig. 2D), respectively, indicating that the lack of detection of hypermethylation was not correlated to cfDNA concentrations (*P* = 0.612, Wilcoxon rank sum test with continuity correction). Methylation status of the ovarian cancer patients (positive, negative, or converted) was not correlated to any of the recorded clinical variables: residual tumor, FIGO, grade, T‐stage, N‐stage, or treatment with PARP inhibitors (Table 1). Surprisingly, hypermethylation of the *BRCA1* gene promoter was also detected in 5/12 (41.7%) patients with germline *BRCA1* mutations. A nonsignificant difference (*P* = 0.376, Welch's two sample *t*‐test) was detected in the median of cfDNA levels between patients who were carriers of germline *BRCA1* mutations (267 ng·mL^−1^) and patients who were negative for germline *BRCA1* mutations (213 ng·mL^−1^). In order to verify our results, all (*n* = 62) cfDNA samples showing signs of hypermethylation were processed by Sanger sequencing. In the MS‐qPCR amplified DNA fragment, five CpG islands are present of which methylation was detected in 97%, 100%, 95%, 89%, and 95% of the sequenced samples, respectively. In each of the samples, methylation of 4 or 5 CpG islands could be confirmed, and one sample showed methylation of three CpG islands only. In all 69 healthy individuals, *BRCA1* promoter hypermethylation was not detected (0/69).

**Fig. 3:**
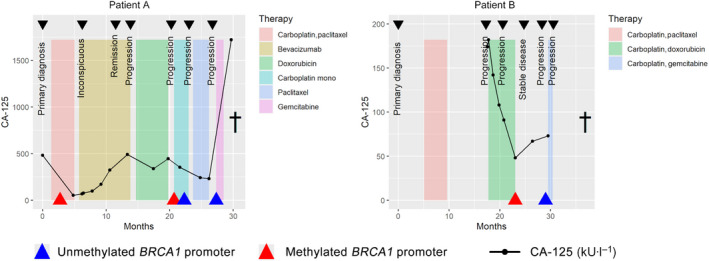
Longitudinal assessment of *BRCA1* promoter methylation status in two cases. Methylation status of the *BRCA1* promoter was assessed in patients during the course of disease until death (†). Clinically recorded data were the systemic therapy (here, doxorubicin is ‘PEG‐liposomal doxorubicin’), CA‐125 (kU·L^−1^), and results from computed tomography (CT) scan‐based staging. Two examples of a methylation conversion (left and right panel).

For illustrative purpose, Fig. 3 depicts the course of disease of two patients, including the concentration of the tumor marker CA‐125 measured for routine diagnostics, the systemic treatments given, the disease status, and the *BRCA1* gene promoter methylation status. Both patients initially exhibited ovarian cancer with a hypermethylated *BRCA1* gene promoter, however, the status converted to presumably functional *BRCA1* during the course of therapy.

### 3.4 Survival analyses

Because the treatment regiments of all ovarian cancer patients were relatively heterogeneous and because ovarian cancer patients can suffer from multiple relapses (see Fig. 3 as example), the survival analyses were performed using two multivariate models testing two hypotheses of how a tumor develops therapy resistance: through therapy‐induced evolution or by selection (illustrated in Fig. S1). In total, 239 events of progression were recorded during follow‐up with a median of three events per patients (range 1–16), with no difference in number of events between the patients with or without mutated *BRCA1* (*P* = 0.879, Wilcoxon rank sum test) and no difference between patients with methylation, without methylation, or a change in methylation status of the *BRCA1* promoter (*P* = 0.309, Kruskal–Wallis rank sum test), suggesting no bias in the survival analyses due to the number of events in single cases.

#### 3.4.1 Model 1: Dependent survival model—Therapy‐induced evolution

**Fig. 4:**
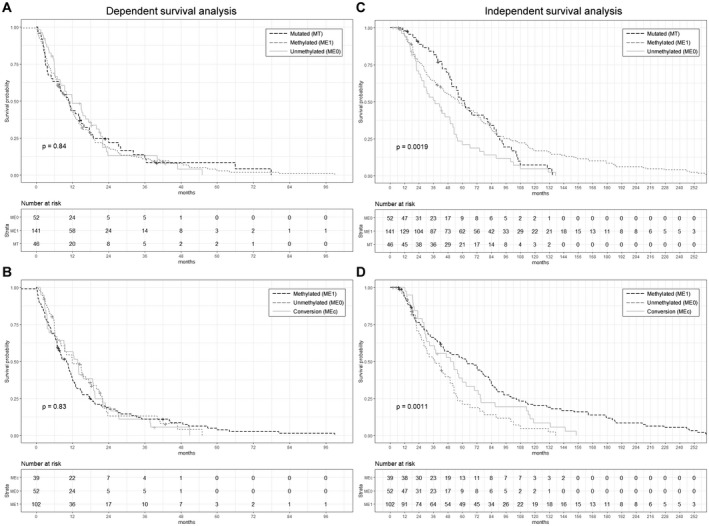
Survival analyses. Kaplan–Meier curves using a multivariate‐dependent survival model on the gap time between successive events of patients showing *BRCA1* gene mutations (A), hypermethylated *BRCA1* promoter (A, B), unmethylated (A, B), and conversion (B). Kaplan‐Meier curves using a multivariate independent survival model on the event time between primary tumor diagnosis and every subsequent progression of disease of patients showing *BRCA1* gene mutations (C), hypermethylated *BRCA1* promoter (C, D), unmethylated (C, D), and conversion (D). *P*‐values were calculated using the log rank test. +, censored.

The first multivariate survival model was applied to test the correlation between the gap time between successive events and *BRCA1* mutation/promoter methylation status, assuming the dependency of subsequent relapses (illustrated in Fig. S1). The median times between events for ovarian cancer patients with *BRCA1* mutations, hypermethylation of the *BRCA1* promoter, and a negative methylation status of the *BRCA1* promoter were 10.9 (95% CI: 7.6–15.9), 10.6 (95% CI: 8.0–12.5), and 12.0 (95% CI: 8.2–18.4) months, respectively (*P* = 0.84, log rank test; Fig. 4A). Excluding the cases with *BRCA1* mutations and separating the methylation positive group into cases with stable positive methylation status and those showing conversion, the median times between events were 10.0 (95% CI: 7.4–11.9) and 13.8 (95% CI: 9.2–19) months, respectively. There was no significant difference between the median gap times between successive events of patients with stable negative, stable positive, or conversion of methylation status (*P* = 0.84, log rank test; Fig. 4B). Progression‐free survival to the first progression only and overall survival were not correlated to methylation status due to the relatively low number of events that require to achieves 80% power at a significant level *P* = 0.15, HR = 0.8.

#### 3.4.2 Model 2: Independent survival model—Therapy‐induced selection

The second multivariate survival model was applied to test the correlation between time from initial diagnosis to each subsequent relapse and *BRCA1* mutation/promoter methylation status, assuming the independency of subsequent relapses and all originating from the primary tumor (illustrated in Fig. S1). Ovarian cancer patients with methylated *BRCA1* promoter detected in cfDNA (median: 57 months; 95% CI: 44.1–72.3) had a comparable survival to ovarian cancer patients with germline *BRCA1* mutations (median: 62 months; 95% CI: 51.0–86.2), but a significantly longer survival than patients with unmethylated *BRCA1* promoter (median 37.5 months; 95% CI: 28.0–52.3; *P* = 0.0019, log rank test; Fig. 4C). The difference between survival of the patients with and without hypermethylated *BRCA1* promoter became more noticeable after excluding the cases with *BRCA1* mutations from the analysis (*P* = 0.0014, log rank test). Interestingly, patients from the methylation positive group who eventually converted to a negative methylation status had a shorter median survival (median: 50.3 months; 95% CI: 30.4–70.0) than the patients who had a stable positive methylation status throughout the whole course of disease (median: 63.8 months; 95% CI: 44.1–81.4), but a better survival than patients with unmethylated *BRCA1* promoter (*P* = 0.0011, log rank test; Fig. 4D). After removal of the nonsignificant clinical variables (FIGO stage, T‐stage, N‐stage, and grade), multivariable analyses showed that methylation status of the *BRCA1* promoter was an independent predictor of survival and that ovarian cancer patients with methylated *BRCA1* promoter had a significant lower risk for disease‐related progression (HR: 0.5614; 95% CI: 0.3774–0.8352; *P* = 0.0044, Cox proportional hazard ratio) as well as patients that showed conversion of the methylation status (HR: 0.6004; 95% CI: 0.3738–0.9644; *P* = 0.0349) as compared to patients with unmethylated *BRCA1* promoter (Table 2). Residual tumor after surgery was as well correlated with a worse survival as compared to who had no residual tumor.

**Table 2:** Cox proportional hazard ratios. Estimated coefficient of hazard ratios (HR) correlated to hypermethylated *BRCA1* promoter and methylation status conversion (reference: unmethylated) and residual tumor after surgery (reference: none), along with 95% CI and *P*‐value. Cases with germline *BRCA1* mutations (*n* = 13) were excluded from this analysis.

Covariate	Coefficient (*b_i_*)	HR [exp(*b_i_*)]	HR 95%CI	*P*‐value
Methylation positive	−0.5102	0.6004	0.3738–0.9644	0.0349
Methylation conversion	−0.5772	0.5614	0.3774–0.8352	0.0044
Tumor rest	1.0895	2.9727	2.0717–4.2656	<0.0001

## 4 Discussion

*BRCA1* promoter hypermethylation was first shown in sporadic breast and ovarian tumors more than 20 years ago [5, 24]. Since then, the development of highly sensitive and specific assays have made minimally invasive, liquid biopsy in oncology possible. Especially in the clinical management of ovarian cancer patients, new assays for real‐time monitoring of therapy response are direly required.

With our liquid biopsy assay, we could show a high sensitivity by detecting down to a single molecule of DNA, minimizing the possibility of failing to detect the tumor's true methylation status. Five CpG sites were investigated to confirm the enrichment of methylated DNA sites, which previously have been documented to be strongly correlated with very low *BRCA1* expression in breast cancer cell lines [5]. Nevertheless, extremely low ctDNA concentrations and the complete absence of tumor DNA in the obtained blood sample may result in a false negative result. Although the latter cannot be excluded and is most likely the case for the two patients in which hypermethylation was detected after a negative plasma sample, cfDNA concentrations were not correlated with methylation status overall, whereas low concentrations of cfDNA have been shown to be associated with better survival [25, 26]. In addition, cfDNA levels have previously shown to be raised in patients with *BRCA1* mutation carriers irrespective of the disease [27]. Furthermore, our data show that a negative methylation status is correlated with a poor progression‐free survival. Taken together, the majority of plasma samples contained enough tumor DNA for a reliable measurement; however, increased blood volumes could be considered. Due to the essential functions of *BRCA1*, hypermethylation of its promoter is not expected to occur in healthy tissue and has thus far not been reported. Because cfDNA is a mixture of DNA originating from practically all regenerating tissues in the body, our data on a group of elderly, cancer‐free, healthy women strongly suggest that hypermethylation of the *BRCA1* promoter only occurs in (pre‐) cancerous cells. Although our data are convincing, a limitation of this study is the lack of positive control for ctDNA presence and should be considered in future studies. Such marker could be *TP53*, the most common mutated gene in high‐grade serous ovarian cancer [1], however, because there are no hotspot mutations in *TP53* or any other known gene in ovarian cancer, a control marker for liquid biopsy may be challenging.

Previously, we showed that a conversion of hypermethylated to wild‐type (unmethylated) *BRCA1* promoter frequently takes place in high‐grade serous ovarian cancer upon recurrence [10]. In the current study, multiple blood samples were obtained from 60% of patients during the course of treatment and a conversion of methylation status could be detected in 24% of methylation positive patients. We hypothesize that methylation conversion is either an active mechanism of resistance as the tumor reactivated *BRCA1* in a response of therapy‐induced DNA double‐strand breaks (e.g., platinum‐based therapy). Alternatively, we postulate that ovarian cancer is a methylomic heterogeneous entity consisting of subclones exhibiting both methylation statuses. Although deactivated DNA repair mechanisms may be advantageous for tumor growth at its early stages, systemic therapy will eventually select for (early‐stage) subclones with active DNA repair mechanisms capable of overcoming chemotherapy. Further in‐depth analyses on tumor methylomic evolution will confirm these hypotheses. Such studies may include ultra‐deep sequencing of the primary tumor to discover minor subclones that may grow out in later stages of the disease as our laboratory has shown to happen in colorectal cancer [28]. Although more data with a bigger cohort are required to confirm our findings, our data suggest that methylation conversion shortens progression‐free survival after initial response and thereby potentially decreasing the median overall survival times of all patients that are initially diagnosed with hypermethylation of the *BRCA1* promoter. Furthermore, in our survival analyses, we used a multivariate model, assuming that relapse is independent of previous relapses. These results may explain why hypermethylation of the *BRCA1* promoter has thus far not been correlated with overall survival and only with progression‐free survival [9].

*BRCA1* promoter hypermethylation in combination with gene mutation has so far been reported only once [29] and has been considered mutually exclusive. We, on the other hand, could show a relatively high frequency of double affected cases. Possible reasons may be temporal and spatial heterogeneity in which differences in *BRCA1* deactivation (LOH or promoter hypermethylation in combination with mutation) throughout the tumor take place during therapy selection over time, or a relatively low sensitivity of techniques in earlier reports compared to our assay. Subclonal investigation of both the genome and methylome is required to answer this question.

A wide range of 5–90% *BRCA1* promoter hypermethylation among ovarian cancer cases has been reported in the past [1, 30, 31, 32, 33, 34]; the wide variability is possibly the consequence of different detection techniques, cohort selection criteria, and/or material sources (i.e., tissue vs. cfDNA). A recent meta‐analysis showed that on average only 16.3% (430/2636) of all reported primary ovarian tumors are showing signs of *BRCA1* promoter hypermethylation, but that hypermethylation is strongly correlated with high‐grade serous carcinomas [9]. Furthermore, in the analyzed literature, Kalachand *et al*. found three different methods for determining promoter methylation status: methylation‐specific PCR, methylation sensitive restriction endonuclease digestion, and genome‐wide methylation arrays; only methylation‐specific PCR was correlated to progression‐free survival (HR: 0.80; 95% CI: 0.66–0.97; *P* = 0.02), which is in line with our data. Taking previously published data together with our results, it can be concluded that the detection of *BRCA1* promoter hypermethylation can be achieved by methylation‐specific PCR. Furthermore, methylation screening could help to identify patients who are likely not to respond to platinum re‐challenge.

## 5 Conclusion

Here, we present the first prospective study in which liquid biopsy was used to assess and monitor the methylation status of the *BRCA1* promoter during platinum‐based therapy in ovarian cancer patients. Our results suggest that hypermethylation of *BRCA1* promoter is correlated with a better survival and that conversion of methylation status is a consequence of therapy‐induced selection rather than cancer evolution. This study opens the avenue for larger clinical studies in which liquid biopsy can be used to monitor the functional status of *BRCA1* by screening for its gene's promoter hypermethylation in real‐time to predict the response to treatment.

## Conflict of interest

The authors declare no conflict of interest.

## Author contributions

KaPr, KlPa, SAJ involved in conceptualization. ME, KaPr, LK, LW, and SAJ performed data curation. ME, LK, and SAJ performed formal analysis. SAJ, VM, and ME involved in funding acquisition. ME, LK, and SAJ investigated the study. ME and SAJ performed methodology and project administration. KaPr, LK, LO‐F, SP, VM, LW, KlPa, and SAJ involved in resources. ME and SAJ performed software. KlPa and SAJ supervised the data. ME and SAJ validated the manuscript, involved in visualization, and wrote original draft. ME, KaPr, LK, LO‐F, SP, VM, LW, KlPa, and SAJ reviewed and edited the manuscript.

### Peer Review

The peer review history for this article is available at https://publons.com/publon/10.1002/1878‐0261.13108.

## Supporting information


**Fig. S1.** Detailed summary of progression models. Two models explain the conversion of *BRCA1* promoter hypermethylation. *BRCA1* promoter hypermethylation is an early event in tumorigenesis. After detection of the primary tumor and multiple rounds of therapy after relapse, the tumor reactivates BRCA1 by evolving and reversing its methylation status and thereby developing therapy resistance (upper panel). Alternatively, multiple subclones may have already developed during tumorigenesis and through multiple rounds of therapy, the most therapy resistant clone eventually survives and thrives (lower panel). The arrows indicate the time from the development to detection of the tumor to be treated, illustrating the need for different statistical models for analysis.

**Table S1.** Detailed summary of the patients' characteristics.

## Data Availability

The data that support the findings of this study are available on request from the corresponding author (s.joosse@uke.de). The data are not publicly available due to privacy or ethical restrictions.
