# Supplementary material for: BRCA1 promoter hypermethylation on circulating tumor DNA correlates with improved survival of patients with ovarian cancer
Source: Mol Oncol. 2021 Oct 12;15(12):3615–25. doi: 10.1002/1878-0261.13108 (PMC8637552; doi:10.1002/1878-0261.13108)
Supplement: Supplementary file 1 — Fig. S1. Detailed summary of progression models. Two models explain the conversion of BRCA1 promoter hypermethylation. BRCA1 promoter hypermethylation is an early event in tumorigenesis. After detection of the primary tumor and multiple rounds of therapy after relapse, the tumor reactivates BRCA1 by evolving and reversing its methylation status and thereby developing therapy resistance (upper panel). Alternatively, multiple subclones may have already developed during tumorigenesis and through multiple rounds of therapy, the most therapy resistant clone eventually survives and thrives (lower panel). The arrows indicate the time from the development to detection of the tumor to be treated, illustrating the need for different statistical models for analysis. [file MOL2-15-3615-s001.pdf]

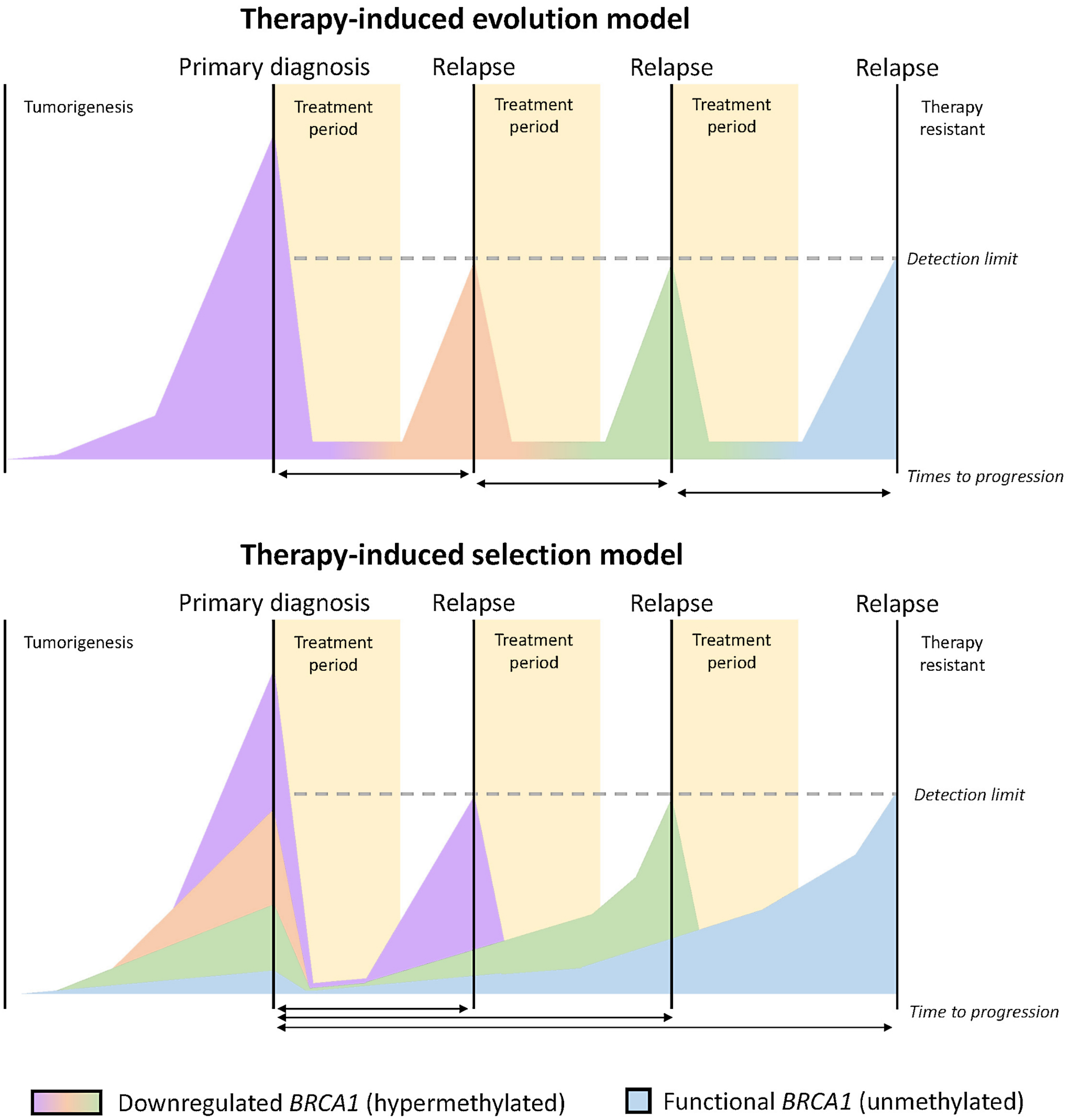

Therapy-induced evolution model
Primary diagnosis
Relapse
Relapse
Relapse
Tumorigenesis
Treatment period
Treatment period
Treatment period
Therapy resistant
Detection limit
Times to progression
Therapy-induced selection model
Primary diagnosis
Relapse
Relapse
Relapse
Tumorigenesis
Treatment period
Treatment period
Treatment period
Therapy resistant
Detection limit
Time to progression
Downregulated BRCA1 (hypermethylated)
Functional BRCA1 (unmethylated)
